# Supplementary material for: Miltefosine increases macrophage cholesterol release and inhibits NLRP3-inflammasome assembly and IL-1β release
Source: Sci Rep. 2019 Jul 31;9:11128. doi: 10.1038/s41598-019-47610-w (PMC6668382; doi:10.1038/s41598-019-47610-w)

Miltefosine increases macrophage cholesterol release and inhibits NLRP3-inflammasome assembly and IL-1β release.

Amanda J Iacano[1], Harvey Lewis[1], Jennie E Hazen[1], Heather Andro[1], Jonathan D Smith[1,2*], and Kailash Gulshan[1*]

SI Materials and Methods.

Materials: The RAW264.7 cells were from ATCC and were cultured in DMEM +10% FBS + penicillin G sodium (100 U/ml) and streptomycin (100 µg/ml). THP-1 Cells were cultured in RPMI 1640 medium supplemented with 10 mM HEPES, 10 % FBS, penicillin G sodium (100 U/ml), streptomycin (100 µg/ml) and supplemented with 0.05 mM 2-mercaptoethanol. The THP-1 cells were differentiated to macrophages using phorbol 12-myristate 13-acetate (Sigma P8139). Miltefosine and 18:1 NBD-PS were obtained from Avanti polar and methyl-β-cyclodextrin is obtained from Sigma Aldrich. ^{3}H-cholestreol (NET13900) was obtained from Perkin- Elmer. Antibodies were purchased from various sources (see table below). MitoSox reagent, Image-iT ™ TMRM reagent, and Alexa FluorTM555 Phalloidin were purchased from Invitrogen.

Novus Bio	ABCA1	NB400-05
Cell signaling	GAPDH (14C10)	2118
	NLRP3 (D4D8T)	15101
	NF-κB p65 (C22B4)	4764
	P-AMPK (The172) (40H9)	2535S
	AMPK Ab	2532S
	P-ULK1	6888
	ULK1	8054
	P-AKT (Ser473)	4060S

	AKT	9272S
Abclonal	GSDMD	A10164
AdipoGen	Caspase-1 (p20)	AG-20B-0042

Isolation of Bone marrow derived macrophages: All experiments were performed in accordance with protocols approved by the Cleveland Clinic Institutional Animal Care and Use Committee (ICUC). WT C57BL/6 or C57BL/6 *ApoE*$^{-/-}$ mice were maintained on chow diet and sacrificed at 16 weeks of age. Femurs were collected to isolate and culture bone marrow macrophages using conditioned L-cell media. Mice were euthanized by CO_2 inhalation and femoral bones were removed. The marrow was flushed out of the bones into a 50 ml sterile tube using a 10 ml syringe with a 26 gauge needle filled with sterile DMEM. Cells were centrifuged for 5 min at 1,800 rpm at 4°C, followed by two washes with sterile PBS. The cells were resuspended in sterile-filtered BMDM growth media (DMEM with 7.6% fetal bovine serum, 15% L-cell conditioned media, and 0.76% penicillin/streptomycin mixture) and plated in 10 cm culture dishes and incubated at 37°C for 14 days. Cell media was replaced every 2–3 days for 2 weeks. The cells were routinely visualized under microscope for proliferation and differentiation into confluent BMDMs.

Isolation of total RNA from BMDM cell pellets and RT-PCR analysis: Total RNA was isolated from the cell pellets using RNeasy Mini Kit (Qiagen Inc. Valencia, CA). One µg of total RNA was reverse transcribed using the iScript cDNA kit (Biorad). Real-time PCR amplification was performed using TaqMan and gene-specific primers in a StepOnePlus™ real time PCR machine (Applied Biosystems) in triplicate. The relative amount of target mRNA was determined using the cycle threshold (Ct) method by

normalizing target mRNA Ct values to that of β-actin. The Fold induction ratios were calculated relative to control sample for each group using the formula $2^{-\Delta\Delta Ct}$.

The reagents were obtained from Qiagen, Biorad, and ThermoFisher (see table below).

Qiagen	RNEasy MiniKit	74104
Qiagen	Rnase-free Dnase set	79254
Biorad	iScript cDNA kit	170-8891
ThermoFisher	2x TaqMan	4304437
ThermoFisher	Nlrp3	Mm00840904
	Casp1	Mm00438023
	iL1B	Mm00434228
	Gsdmd	Mm00509958
	Actb	Mm02619580

Cholesterol release assay: The cholesterol efflux assays were performed in HEK293-Abca1-GFP cells, BHK cells, or RAW 264.7 murine macrophages. The cells were plated in 24-well plates at a density of 300-400,000 cells per well. The cells were labeled with ^{3}H cholesterol in 1% FBS in DMEM for 24h. The labeled cells were induced for ABCA1 expression for 16h with 0.3 mM 8Br-cAMP (for RAW264.7 cells) or with 10nM Mifepristone (for BHK cells). Inducers were included in the media during subsequent assays. ABCA1 expression was confirmed by western blot using the AC10 antibody. The cells were washed with serum free media and chased for indicated time period in serum-free DMEM in the presence or absence of indicated treatments. The radioactivity in the chase media was determined after brief centrifugation to pellet any residual debris. Radioactivity in the cells was determined by extraction in hexane:isopropanol (3:2) with the solvent evaporated in a scintillation vial prior to counting. The percent cholesterol efflux was calculated as 100 × (medium dpm) / (medium dpm + cell dpm).

Lipid-Raft quantification: The lipid-raft were visualized under fluorescent microscope and quantified using flow-cytometry assay. RAW cells were treated with 7.5 μg/ml Miltefosine at 37°C for 16h. For imaging, cells were treated with 1 μg/ml Alexa647-CT-B labeled cells and images were captured using epifluorescent microscopy. For quantification, the cells were washed twice with PBS and gently scraped in 1.5 ml PBS. The Alexa647-CT-B (1μl of 1.5 mg/ml stock in PBS was added to 1.5 ml cells) was added at final concentration of 1 μg/ml to cells in 5 ml polystyrene round-bottom tubes (12 X 75 mm). The samples were subjected to Flow analysis with BD Biosciences LSRII cytometer using laser (Alexa 647 (Red) Ex: 639, Em: 650-670, Filter 660/20) and data was analyzed by Flowjo software.

Cell-surface PS and NBD-PS translocation: To measure cell surface PS, cells were cultured in 6 well plates in growth media, washed twice with PBS, and were resuspended in 500 μl Annexin V binding buffer plus 1 μl of Annexin V-Cy5 (Biovision). The samples were incubated at room temperature for 5 minutes in the dark. Flow cytometry analysis was performed using a BD Biosciences LSRFortessa cytometer using a 639 nm excitation laser and emission at 650-670 nm; and, data was analyzed using Flowjo software. To measure the inward translocation of various NBD labeled PS, RAW cells were incubated with phenol-red free DMEM containing 25 μM NBD-PS for 15 min at RT. The media was then replaced with PBS. The cells were gently scraped and were subjected to flow cytometry analysis first without sodium dithionite to calculate total NBD fluorescence, and then 5 min after the addition of 30 μl of a freshly prepared 1M sodium dithionite solution to determine the fraction of the signal resistant to

quenching (translocated signal). Flow cytometry analysis was performed as above using a 488 nm laser for excitation and emission at 505-525 nm.

Western blotting: RAW264.7 cells or BMDMs were grown treated as indicated. The PBS-washed cell pellet was lysed in NP40 lysis or MPER lysis buffer. After discarding the nuclear pellet, the protein concentration was determined using the BCA protein assay (Pierce). 10-50 µg of cell protein samples were resolved on Novex 4-20% Tris-Glycine Gels (Invitrogen) and transferred onto polyvinylidene fluoride membranes (Invitrogen). Blots were incubated sequentially with 1:1000 rabbit polyclonal antibody raised against Nlrp3 (cell signaling), or 1:5000 rabbit polyclonal antibody raised against P-AMPK (cell signaling), or 1:1000 rabbit polyclonal antibody raised against IL1-b (cell signaling), 1:1000 rabbit polyclonal antibody raised against Gasdermin D, 1:1000 rabbit polyclonal antibody raised against ASC1. The signal was detected with an enhanced chemiluminescent substrate (Pierce). For LC3 western blot, RAW264.7 cells were incubated with or without 7.5 µM Miltefosine for 16 h, indicated wells received 30 µM chloroquine during the last 2 h of incubation. Blots were sequentially incubated with 1:1,000 rabbit polyclonal antibody raised against LC3B and 1:10,000 horseradish peroxidase-conjugated goat anti-rabbit as described above. GAPDH was used as a loading control.

Total and cell surface ABCA1 levels: RAW 267.4 cells were cultured in DMEM with 10% FBS and were treated with 300 µM 8Br-cAMP to induce ABCA1 for 16 hr. Cells were incubated with incubated with or without 7.5µM Miltefosine for 4h. The cells were then incubated for 30 minutes on ice with phosphate-buffered saline (PBS) containing 1 mg/mL sulfo-NHS-biotin (Pierce). The PBS-washed cell pellet was lysed in 200 µl of

lysis buffer (150 mM sodium chloride, 5 mM EDTA, 50 mM Tris-phosphate pH 8.0, 1% NP40, and 10% protease inhibitor). Cell-surface ABCA1 was determined by purifying biotinylated proteins by overnight incubation of 250 ug of cell protein with 75 uL of UltraLink Plus Immobilized Streptavidin gel (Pierce) at 4°C. The beads were spun down, washed, and resuspended by boiling in 100 μl 1x NuPage LDS Sample buffer (Invitrogen) and 20 μg of total protein were run on NuPAGE 3-8% Tris-Acetate gels (Invitrogen) and transferred onto polyvinylidene fluoride membranes (Invitrogen). Blots were incubated sequentially with 1:500 mouse monoclonal antibody raised against ABCA1 (AC10) and 1:15 000 horseradish peroxidase-conjugated goat anti-mouse secondary antibody (Biorad). The signal was detected with an enhanced chemiluminescent substrate (Pierce).

Cellular immunofluorescence and flow cytometry antibody binding assays: RAW264.7 cells or BMDMs were grown in 4-chamber slides in indicated growth media. Cells were washed with 1xPBS, fixed with paraformaldehyde, and then incubated with permeabilization solution (1xPBS/0.2% Triton X-100) for 10 min at RT. Cells were washed with 1xPBS three times and blocked with normal goat serum (1xPBS/1%BSA/5% normal goat serum) at 37°C for 30 min. The cells were probed with p62 antibody and Alexa 647 labeled secondary antibody. For LC3 staining, RAW264.7 cells were transfected with LC3-GFP (Addgene) using lipofectamine LTX according to manufacturer protocol (Life Technologies). Cells were treated with or without 7.5 μM Miltefosine for 16 h. For ASC staining, BMDM from WT C57BL6 or C57BL6 apoE$^{-/-}$ KO mice were treated ± 5 μM Miltefosine for 16 h or ± 1mM cyclodextrin for 2h. Cells were washed with 1xPBS and fixed with 100% ethanol, followed by staining with anti-ASC

antibody and Alexa 568 labeled secondary antibody. All images were captured using an Olympus IX51 inverted epifluorescent microscope, Olympus LUCPlanFI ×40/0.6 lens, with a Q-Image EXi aqua camera and Olympus cellSens Dimension version 1.7 software. Post-imaging processing was performed in Adobe Photoshop CS2. For flow cytometry based analysis of TLR4 antibody binding, BMDMs were treated ± 5 μM Miltefosine for 16 h, followed by priming with LPS for 4h. The cells were fixed with methanol free fixing solution (Electron Microscopy Solutions, Cat#15710) and blocked with anti-goat serum and incubated with either mouse TLR4 antibody (Santa Cruz, SC-52962) or control IgG for 2h in ice. The cells were washed with PBS-goat serum and incubated with Alexa 568 labeled goat anti-mouse secondary antibody (ThermoFisher) for 1h at RT. The cells were collected by gentle scrapping and subjected to flow cytometry analysis.

Mitochondrial ROS production and membrane potential measurement: Mitochondrial ROS levels were determined in control or Miltefosine treated BMDMs that were primed with 1ug/ml LPS for 4h, followed by staining with MitoSOX™ Red Mitochondrial Superoxide Indicator (ThermoFisher) using manufacturer's protocol. Cells were either visualized by fluorescent microscopy or subjected to flow-cytometry analysis. Mitochondrial membrane potential measurements were determined in control or Miltefosine treated BMDMs that were primed with 1ug/ml LPS for 4h, followed by staining with Image-iT™ TMRM Reagent (ThermoFisher) using manufacturer's protocol. Cells were visualized by fluorescent microscopy or subjected to flow-cytometry analysis.

Non-standard abbreviations and acronyms:
ABCA1, ATP-binding cassette transporter A1
AIM2, absence in melanoma 2
AMPK, AMP-activated protein kinase
apoA1, apolipoprotein A-I
ASC, apoptosis-associated speck protein containing a CARD (Caspase activation and recruitment domain)
Gsdmd; Gasdermin D
HDL-C, high-density lipoprotein-cholesterol
IL-1β, Interleukin 1-beta
NLRP3, NOD-like receptor family pyrin domain-containing 3
NF-kB, nuclear factor kappa-light-chain-enhancer of activated B cells
PC, phosphatidylcholine
PIP2, phosphatidylinositol 4,5-bisphosphate
PS, phosphatidylserine
TLR, toll like receptor

Supplementary Figure Legends

Figure S1: Miltefosine increased ABCA1 mediated cholesterol efflux. A) The LDH release assay showing % LDH release in RAW264.7 macrophages as compared to control cells (mean ± SD, N=3, ***, p<0.001 by ANOVA Bonferroni posttest, all columns compared to control column). ***B)*** The LDH release assay showing % LDH release in BMDMs as compared to control cells(mean ± SD, N=3, ***,p<0.001 by ANOVA Bonferroni posttest, all columns compared to control column). ***C)*** BHK cells were labeled with ^{3}H-cholesterol and pretreated with or without Mifepristone to induce ABCA1 expression. Cholesterol release to media was performed for 4h at 37°C in serum-free DMEM without addition of acceptor containing either vehicle or 7.5 μM Miltefosine. Values are % cholesterol efflux, mean ± SD, N=5, **p<0.01, **** p<0.0001 by ANOVA Bonferroni posttest comparing all columns with control. ***D)*** THP cells were differentiated into macrophages by addition of100nM PMA to media. THP cells were labeled with ^{3}H-cholesterol and pretreated ± 1 μMT0901317 (Sigma, Cat #T2320) compound to induce ABCA1 expression. Cholesterol release to media was performed for 4h at 37°C in serum-free DMEM ± lipid-free apoA1 (5μg/ml) containing either vehicle or 7.5 μM Miltefosine. Values are % cholesterol efflux, mean ± SD, N=5, **p<0.01, **** p<0.0001 by ANOVA Bonferroni posttest comparing all columns with control. ***E,F)*** RAW cells were labeled with ^{3}H-cholesterol and pretreated ± 300 μM 8Br cAMP to induce ABCA1 expression. Cholesterol release to media was performed for either 1h (*E*) or 6h (*F*) at 37°C in serum-free DMEM ± lipid-free apoA1 (5μg/ml) containing either vehicle or 7.5 μM Miltefosine. Values are % cholesterol efflux, mean ± SD, N=3, **p<0.01, **** p<0.0001 by ANOVA Bonferroni posttest comparing all columns with respective controls. ***G)*** The WT ABCA1-GFP and double mutant ABCA1-GFP isoform expression measured by flow cytometry analysis (Values are GFP intensity, mean ± SD, N=4, different letters above the bars show p<0.0001 by two-tailed t-test.

Figure S2: A) Miltefosine increased microparticle generation and inhibited basal AKT phosphorylation. RAW cells were treated with ± 7.5 μM Miltefosine for 4h at 37°C. The media was collected and cleared of cell debris by centrifugation at 5K rpm for 5 min. The supernatant was spun down at 15K for 30 min. The pellet was suspended in sterile PBS (filtered with 0.22 μM filter) and microparticle were detected using Zetaview particle analyzer. Values are number of microparticles per ml, mean ± SD, N=3, different letters above the bars show p<0.001 by t-test. ***B)*** Miltefosine inhibited basal AKT phosphorylation with or without insulin treatment. RAW cells were treated ± 5 μM Miltefosine for 16h at 37°C and were tested for AKT phosphorylation ± 1μg/ml insulin (R & D systems, #7544) for 20 min. Cell extracts were prepared in MPER lysis buffer containing phosSTOP (Roche). Western blot analysis was carried out using anti-Phospho-AKT and AKT antibodies. ***C)*** P-AKT/AKT ratio using band densitometry (values are band intensity, mean ± SD, N=3, different letters above the bars show p<0.01 by ANOVA Bonferroni posttest (For N=3, 3rd lane is not shown in blot). ***D)*** The raw data from flow cytometry experiment for figure 2C, showing increased binding of

Annexin-Cy5 in cells treated with Miltefosine alone vs. control. Cells expressing ABCA1 showed increased binding vs. control while cells expressing ABCA1 and treated with Miltefosine showed further increase in Annexin Cy binding.

Figure S3: RAW264.7 cells stably transfected with 2X-PH-PLCeGFP reporter plasmid ± 7.5 μM Miltefosine treatment and DAPI counterstained. Control cells showed plasma membrane localization while cells treated with Miltefosine showed PIP2 mislocalization.

Figure S4: Miltefosine induced autophagy. Full length blot of Fig. 4B with high exposure (***S4A***) and low exposure (***S4B***) showing control, chloroquine alone, Miltefosine alone and Chq+Miltef samples. ***C)*** Full graph for figure 4C showing Miltefosine alone sample along with others shown in main ***Fig. 4C.*** ***D) Miltefosine decreased lipid droplets.*** RAW cells were loaded with 100μg/ml AcLDL for 24h, followed by 4 hr chase ± 7.5 μM Miltefosine for 4h at 37°C. The cells were then stained with Nile-red and counterstained with DAPI and imaged using fluorescent microscope. ***E, F***) ***Miltefosine induced AMPK and ULK1 phosphorylation.*** RAW cells were treated ± 7.5 μM Miltefosine for 4h at 37°C. The cells extracts were prepared in MPER lysis buffer containing phosSTOP (Roche). Western blot analysis was carried out using anti-phospho-AMPK, anti-phospho-ULK1, AMPK and ULK1 antibodies.

Figure S5: Miltefosine dampens TLR4 signaling pathway. A) Full graph of ***Fig. 5A*** and ***B)*** raw flow-cytometry data showing TLR4 antibody binding.

Figure S6: Miltefosine inhibited Nlrp3 inflammasome assembly. Full images of cropped sections for control cells shown in ***Figure 6A***.

Figure S7: Miltefosine inhibited Nlrp3 inflammasome assembly. Full images of cropped sections for Miltefosine tretaed cells shown in ***Figure 6A***.

Figure S8: Miltefosine did not alter the protein levels of Nlrp3 inflammasome complex. Mouse BMDMs were treated with ± 5 μM Miltefosine for 16h at 37°C. The cells were primed by incubation with ± 1mg/ml LPS at 37°C for 4hrs, followed by either collection of media and western blot analysis of supernatants using Caspase 1 antibody (**A**) or preparation of cell extracts followed by western blot analysis using NLRP3 antibody (***B***), ASC (***C***), Caspase 1 (***D***) along with GAPDH controls.

Figure S9: Miltefosine inhibited Nlrp3 inflammasome assembly. Asc specks and DAPI staining in cyclodextrin treated BMDMs.

Figure S10: Miltefosine altered mitochondrial homeostasis. TMRM staining of mouse BMDMs pretreated ± 5 μM Miltefosine and treated ± 1μg/ml LPS.

Figure S11: Miltefosine altered mitochondrial homeostasis. Mouse BMDMs pretreated ± 5 μM Miltefosine were stained with mitophagy dye (Mitophagy detection kit MD01, Dojindo Molecular Technologies, Inc.)

Figure S1

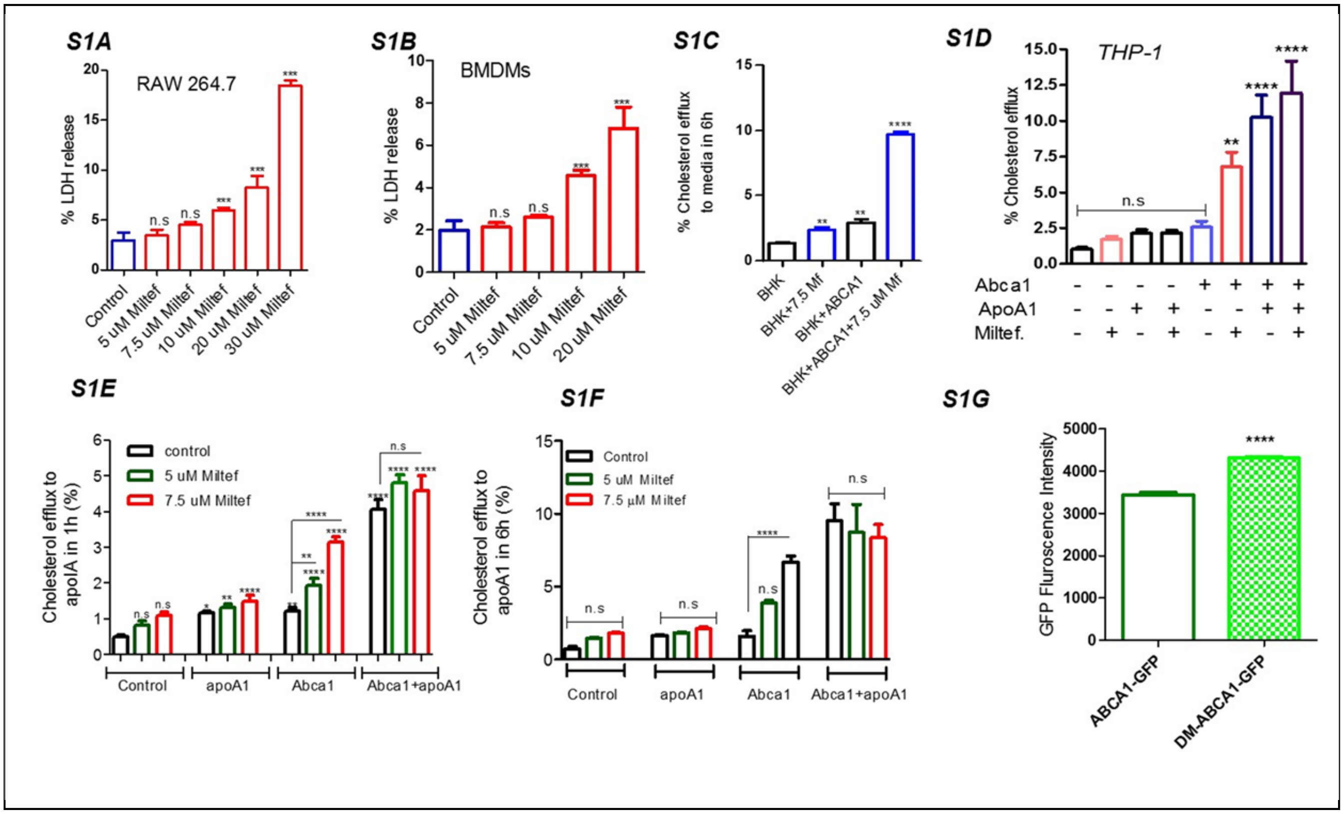
S1A
RAW 264.7
% LDH release
Control
5 uM Miltef
7.5 uM Miltef
10 uM Miltef
20 uM Miltef
30 uM Miltef
S1B
BMDMs
% LDH release
Control
5 uM Miltef
7.5 uM Miltef
10 uM Miltef
20 uM Miltef
S1C
% Cholesterol efflux to media in 6h
BHK
BHK+7.5 Mf
BHK+ABCA1
BHK+ABCA1+7.5 uM Mf
S1D
THP-1
% Cholesterol efflux
Abca1
ApoA1
Miltef.
S1E
Cholesterol efflux to apoA in 1h (%)
control
5 uM Miltef
7.5 uM Miltef
Control
apoA1
Abca1
Abca1+apoA1
S1F
Cholesterol efflux to apoA1 in 6h (%)
Control
5 uM Miltef
7.5 μM Miltef
Control
apoA1
Abca1
Abca1+apoA1
S1G
GFP Fluroscence Intensity
ABCA1-GFP
DM-ABCA1-GFP

Figure S2

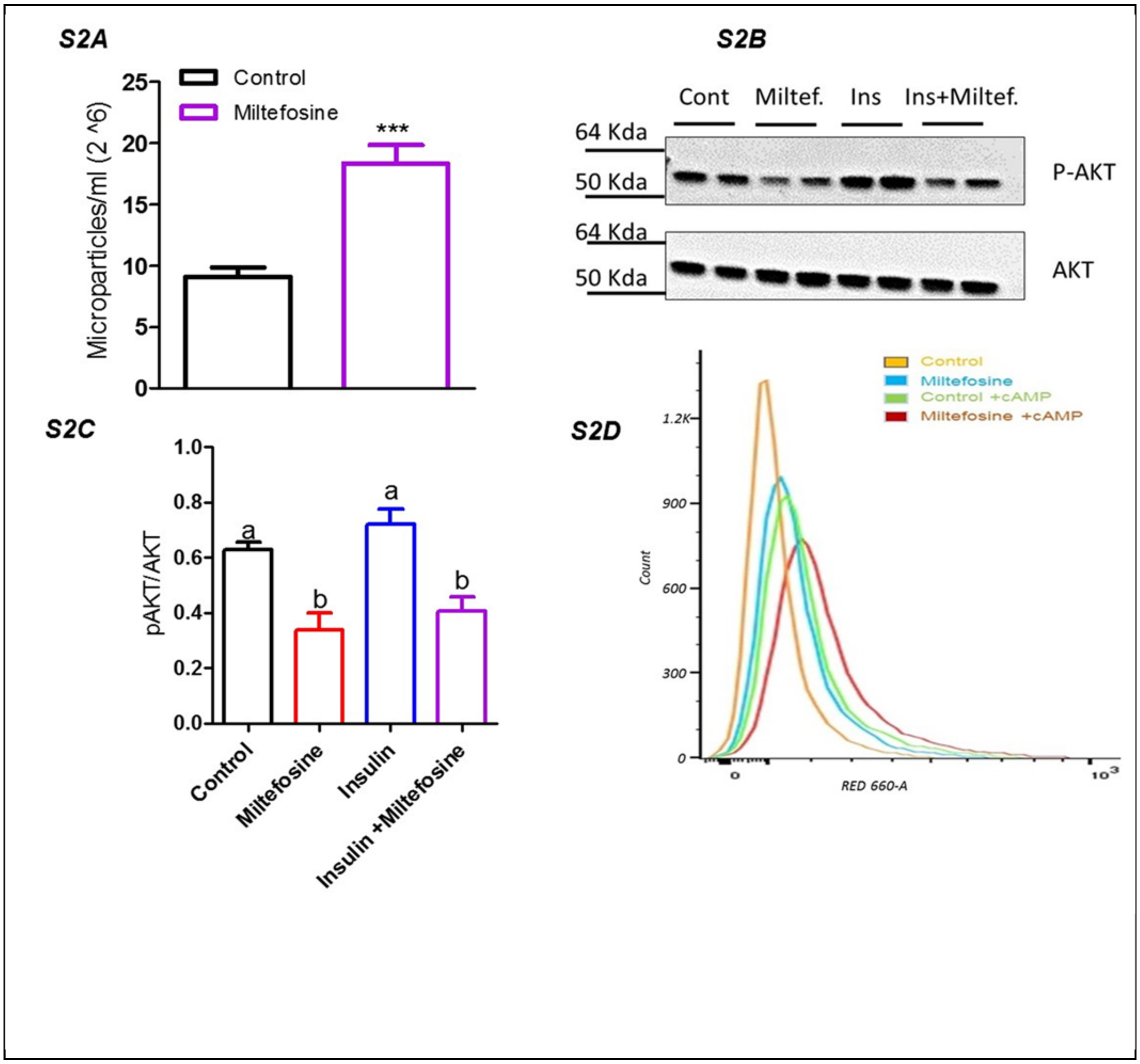

S2A
Control
Miltefosine
Microparticles/ml (2 ^6)
***
S2B
Cont
Miltef.
Ins
Ins+Miltef.
64 Kda
50 Kda
P-AKT
AKT
S2C
pAKT/AKT
a
b
a
b
Control
Miltefosine
Insulin
Insulin +Miltefosine
S2D
Control
Miltefosine
Control +cAMP
Miltefosine +cAMP
Count
RED 660-A

Figure S3

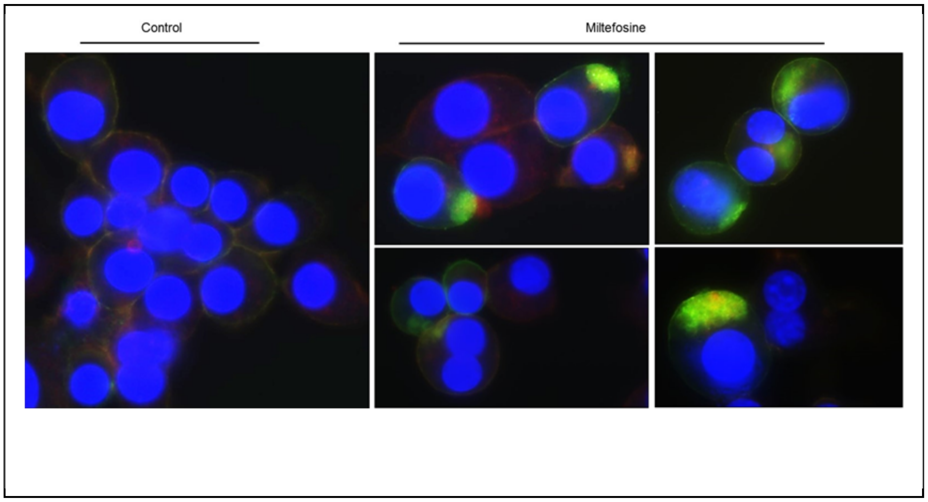

Figure S4

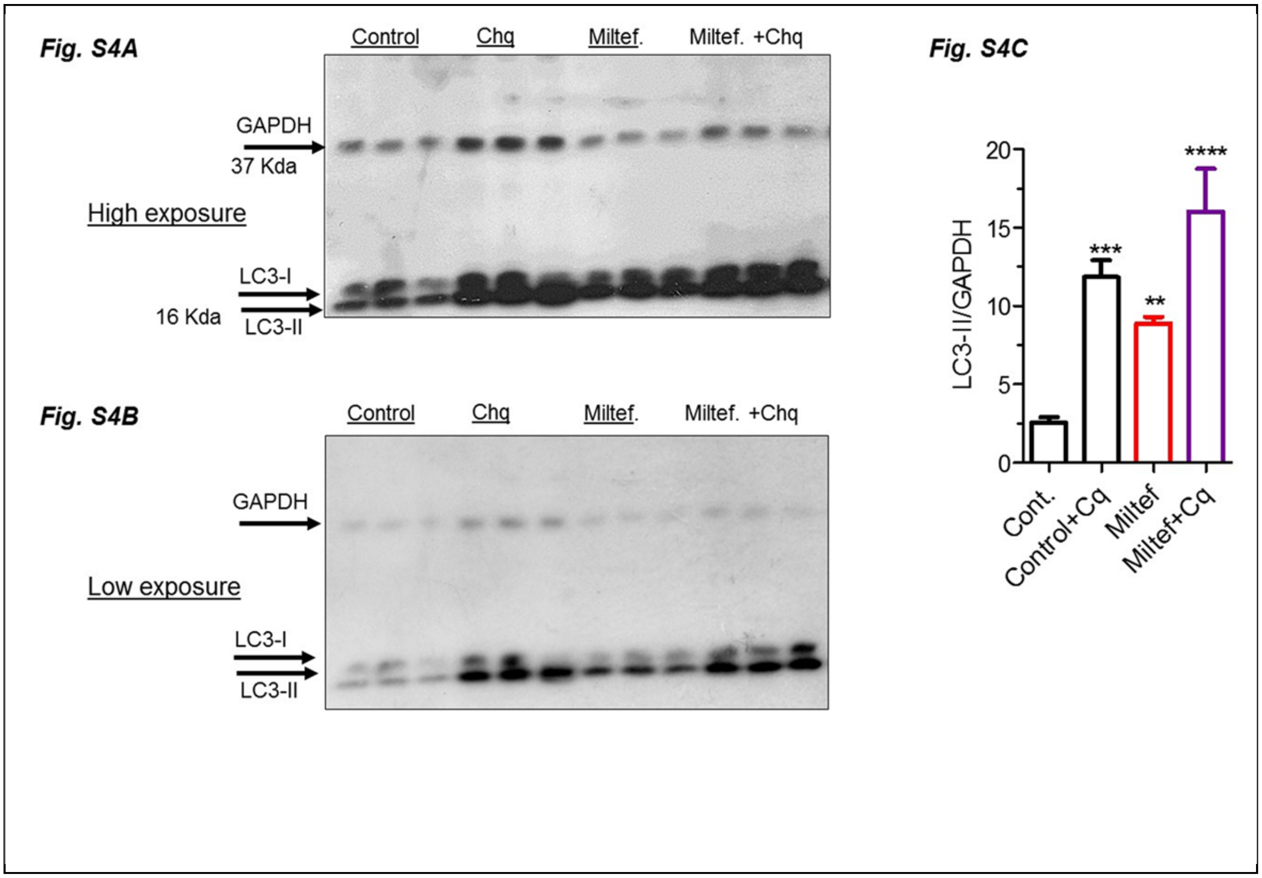

Fig. S4A
Control
Chq
Miltef.
Miltef. +Chq
GAPDH
37 Kda
High exposure
LC3-I
16 Kda
LC3-II
Fig. S4B
Control
Chq
Miltef.
Miltef. +Chq
GAPDH
Low exposure
LC3-I
LC3-II
Fig. S4C
LC3-II/GAPDH
20
15
10
5
0
***
**
****
Cont.
Control+Cq
Miltef
Miltef+Cq

Fig. S4D

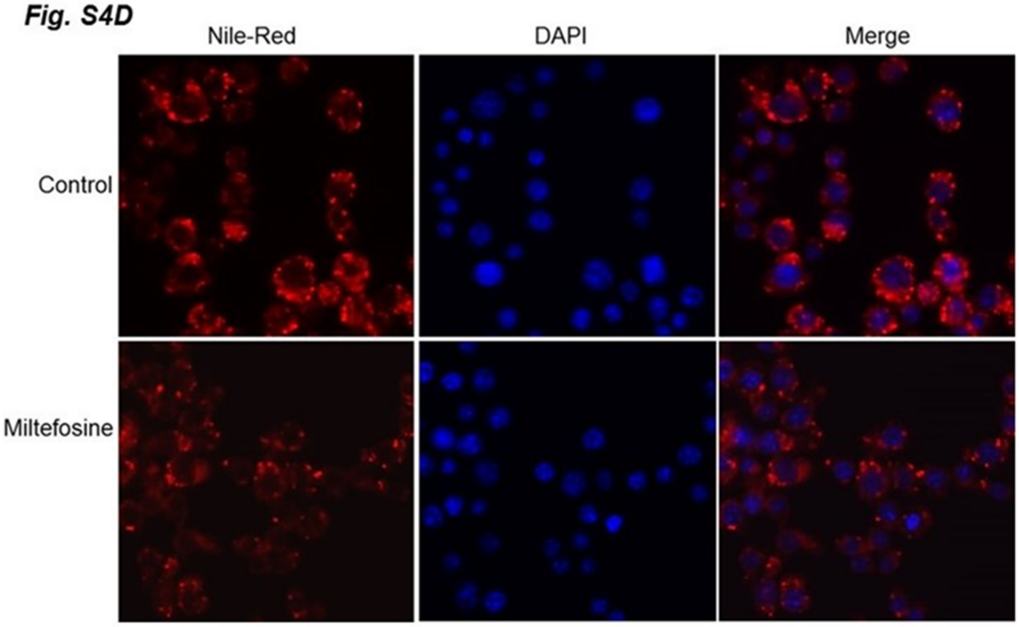

Fig. S4E

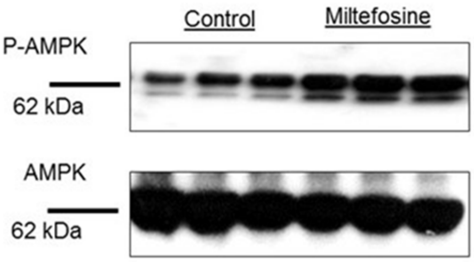

Fig. S4F

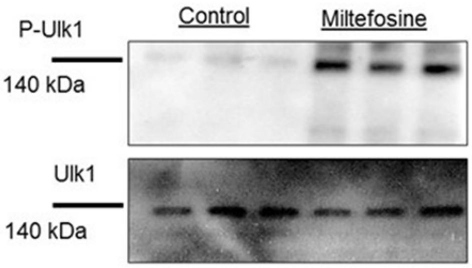

Figure S5

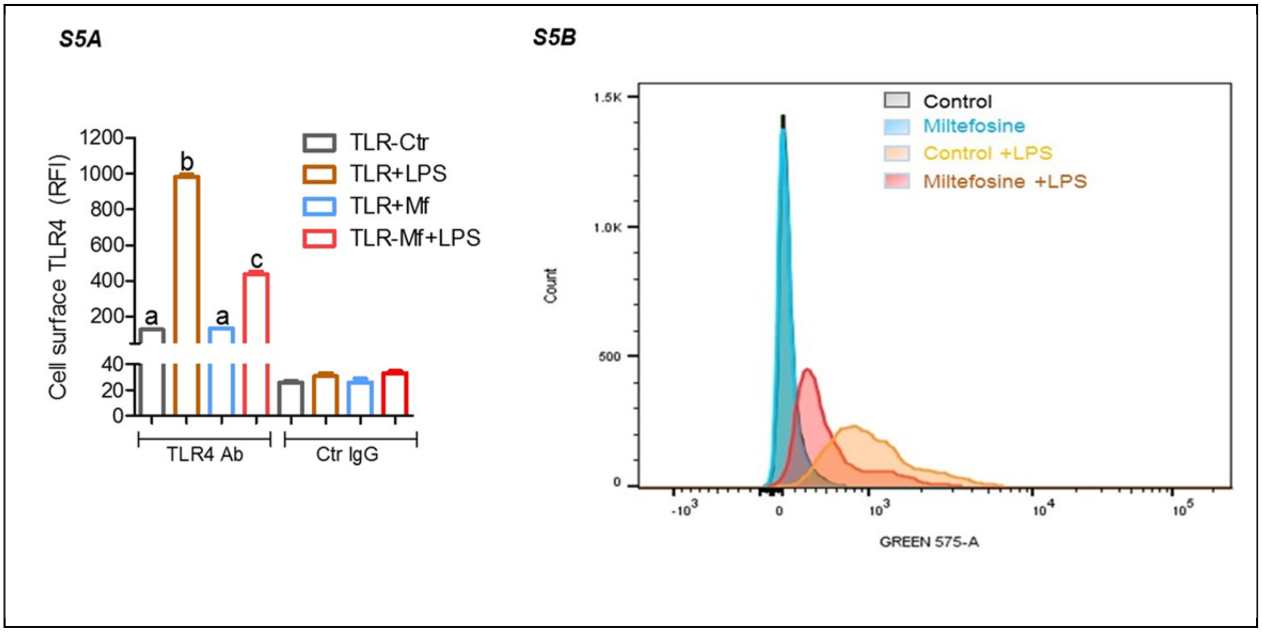

S5A
Cell surface TLR4 (RFI)
1200
1000
800
600
400
200
40
20
0
a
b
a
c
TLR-Ctr
TLR+LPS
TLR+Mf
TLR-Mf+LPS
TLR4 Ab
Ctr IgG
S5B
Control
Miltefosine
Control +LPS
Miltefosine +LPS
1.5K
1.0K
500
0
Count
-10^3
0
10^3
10^4
10^5
GREEN 575-A

Figure S6

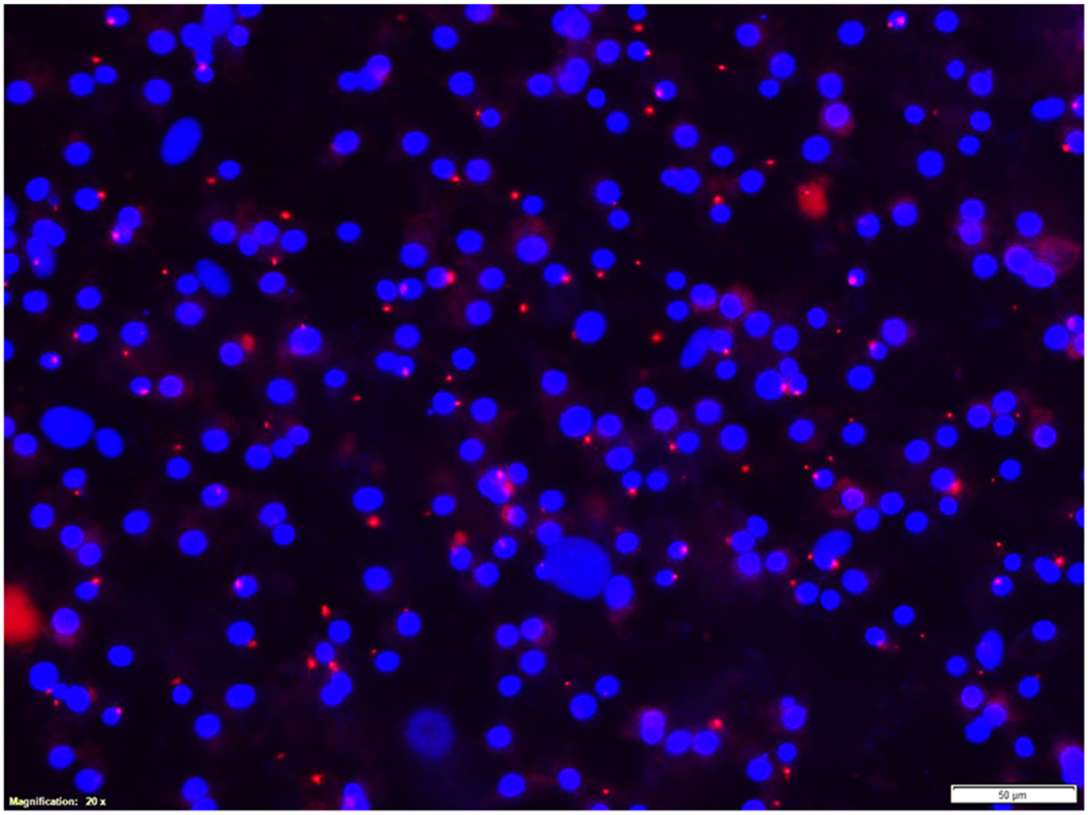

Fig. S7

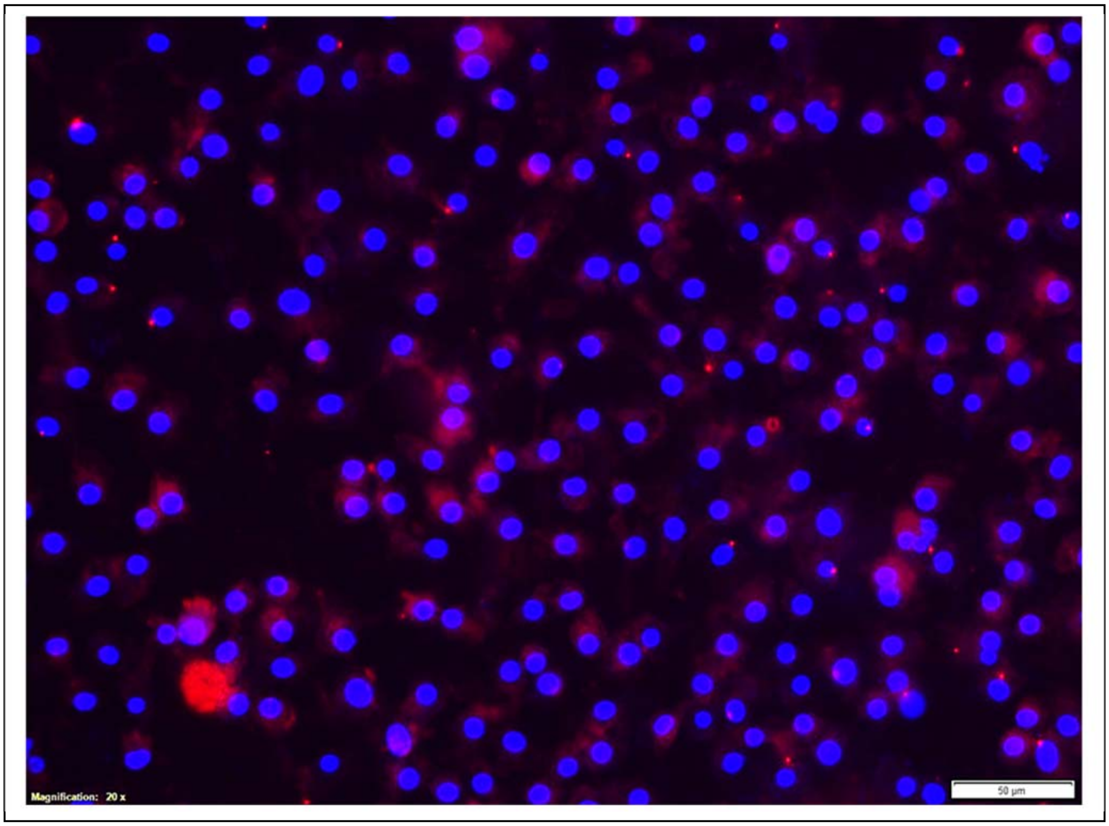

Fig. S8

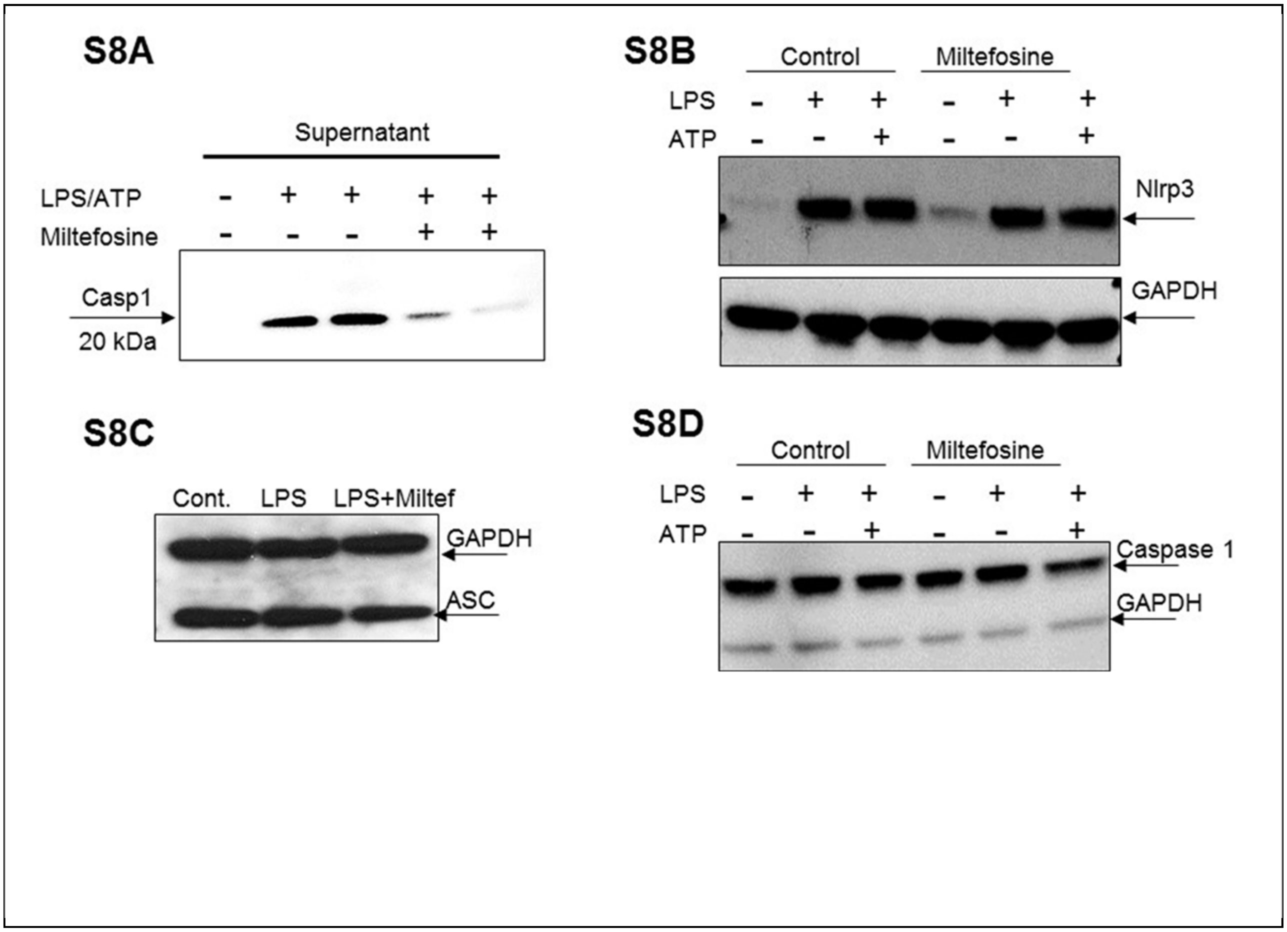
S8A
Supernatant
LPS/ATP - + + + +
Miltefosine - - - + +
Casp1
20 kDa
S8B
Control
Miltefosine
LPS - + + - + +
ATP - - + - - +
Nlrp3
GAPDH
S8C
Cont.
LPS
LPS+Miltef
GAPDH
ASC
S8D
Control
Miltefosine
LPS - + + - + +
ATP - - + - - +
Caspase 1
GAPDH

Fig. S9

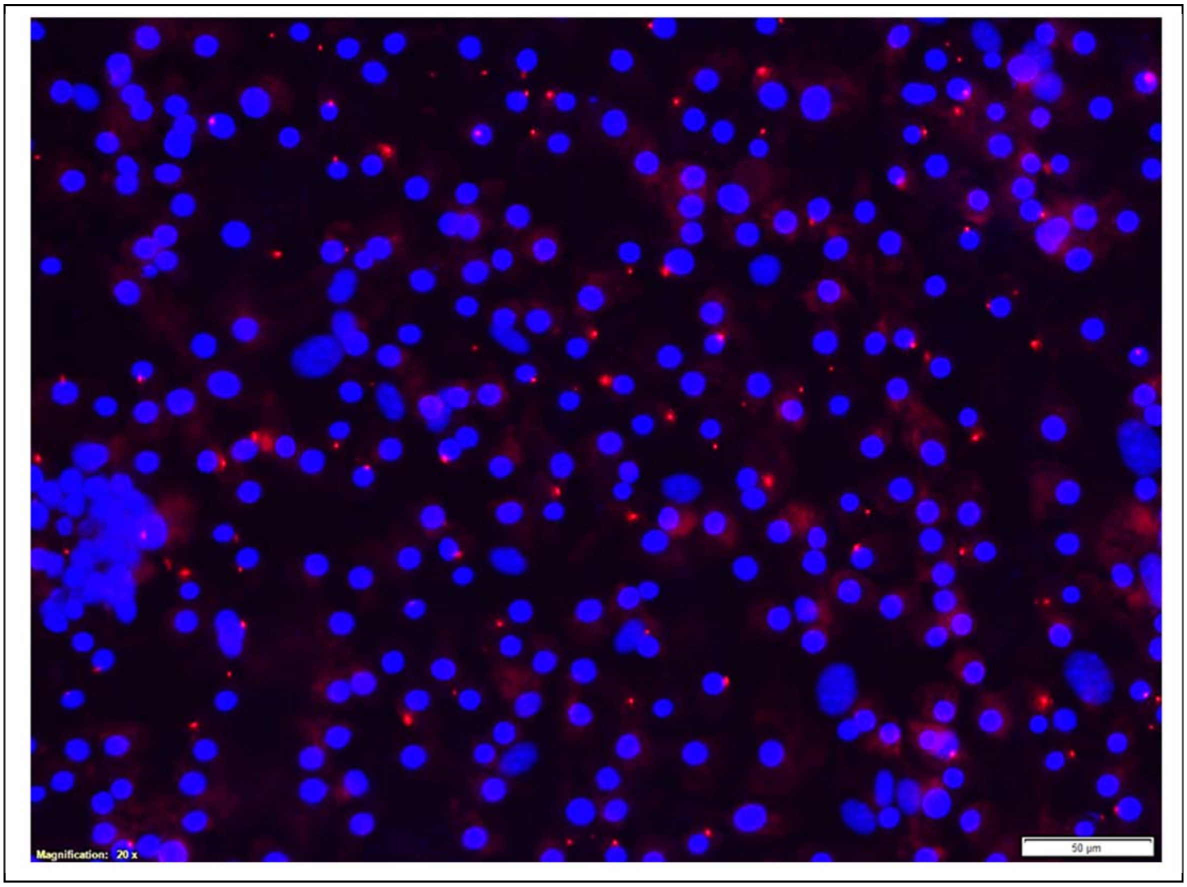

Fig. S10

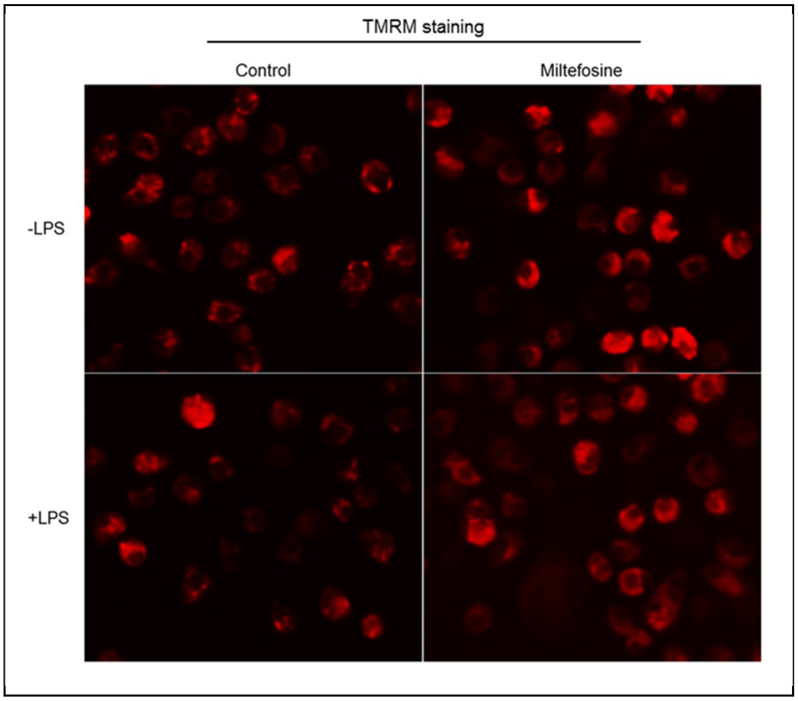

Fig. S11

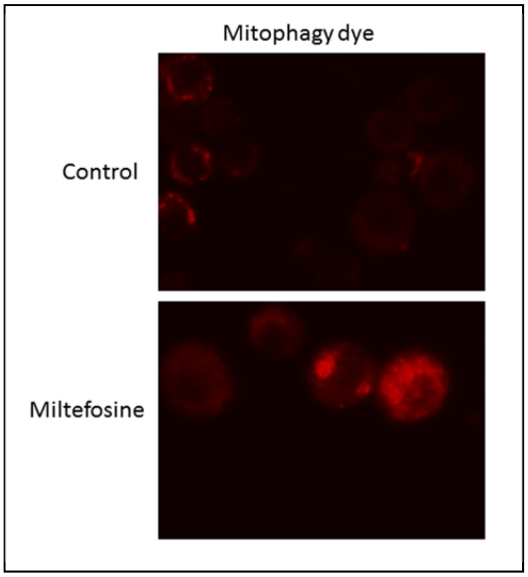

Fig. S12: First four lanes were cropped and were showed in separate boxes in Fig. 1 B.

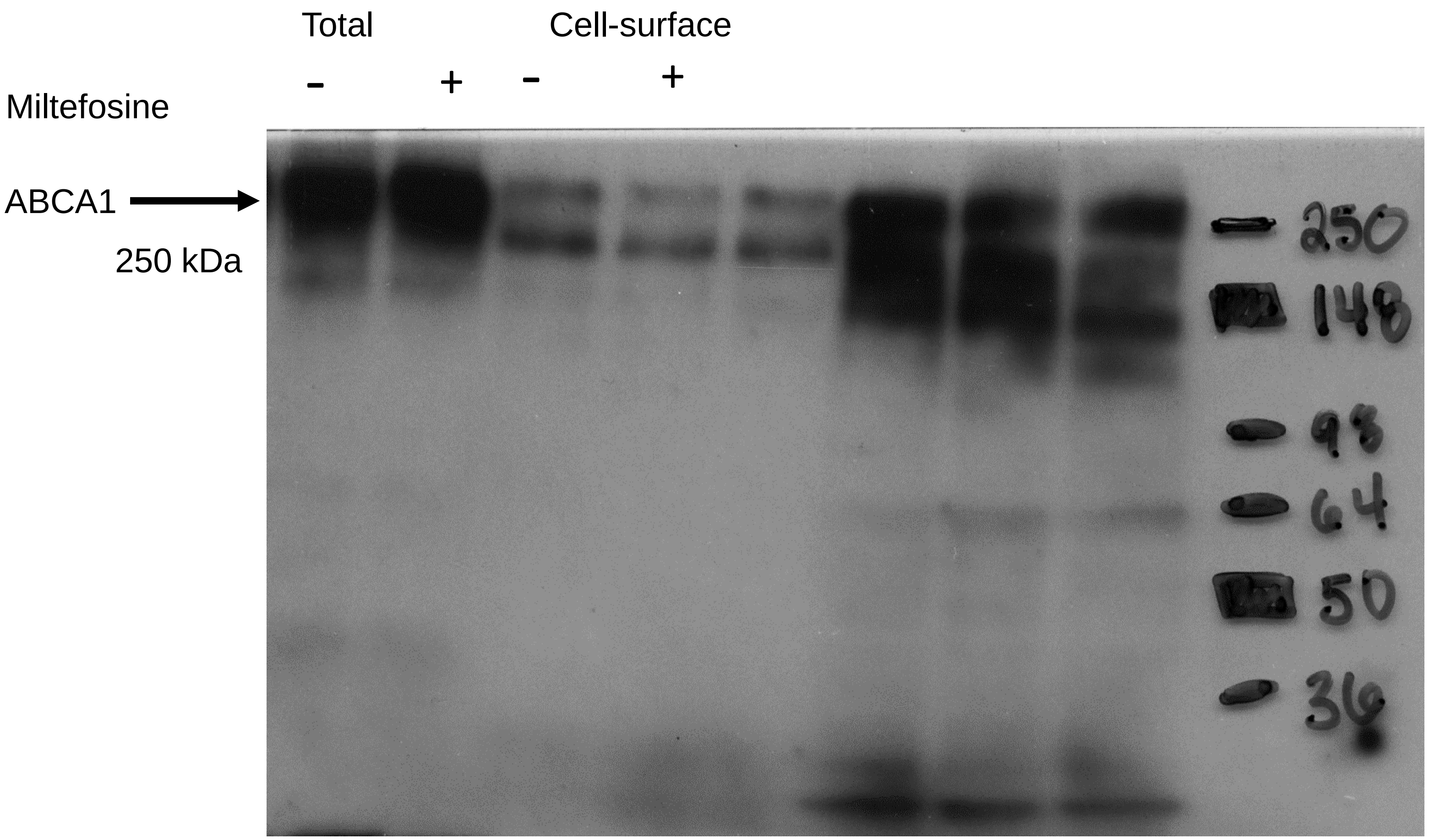

Fig. S13: Full blot Fig. S4A and S4B.

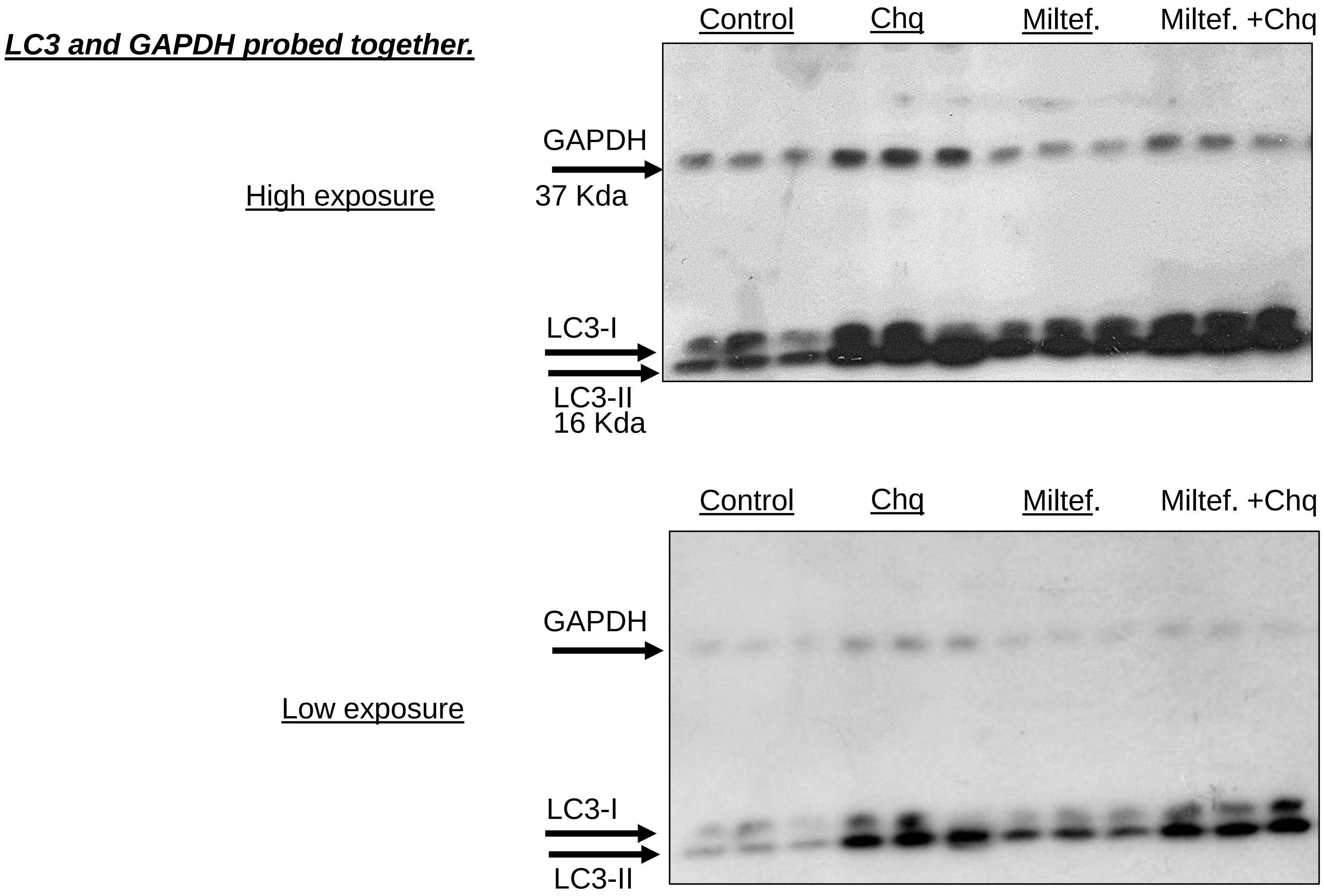

Fig. S14: Full blot of image Fig. 5B. The GAPDH blot was probed along Nlrp3 antibody (same membrane as used for IL-1b).

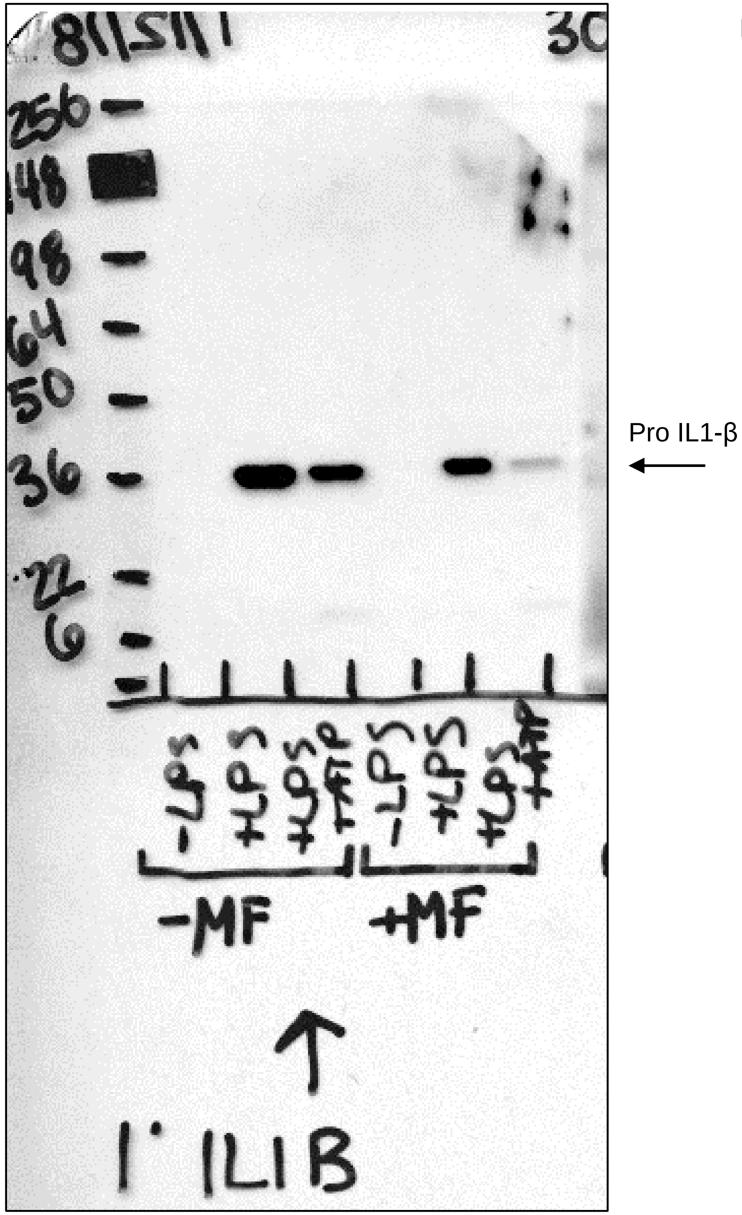

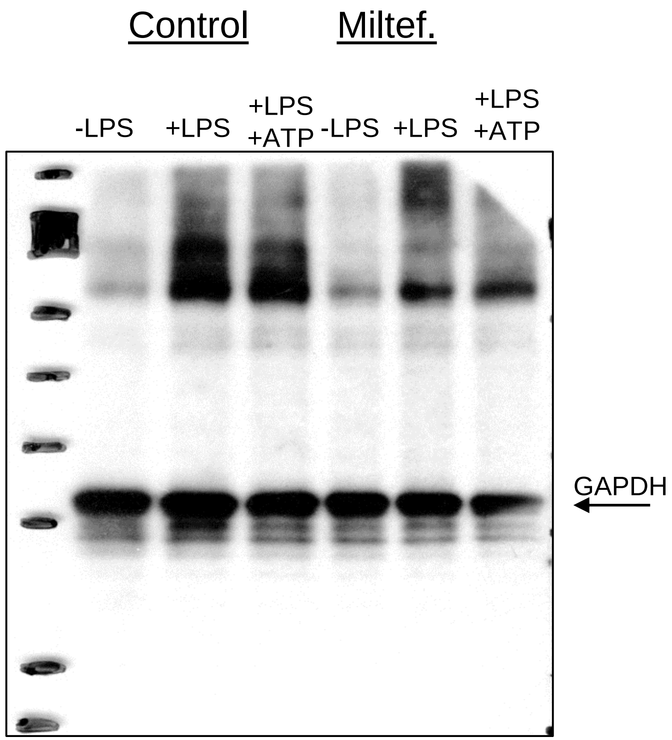

Fig. S15: Full blot of supplementary Figure S2B.

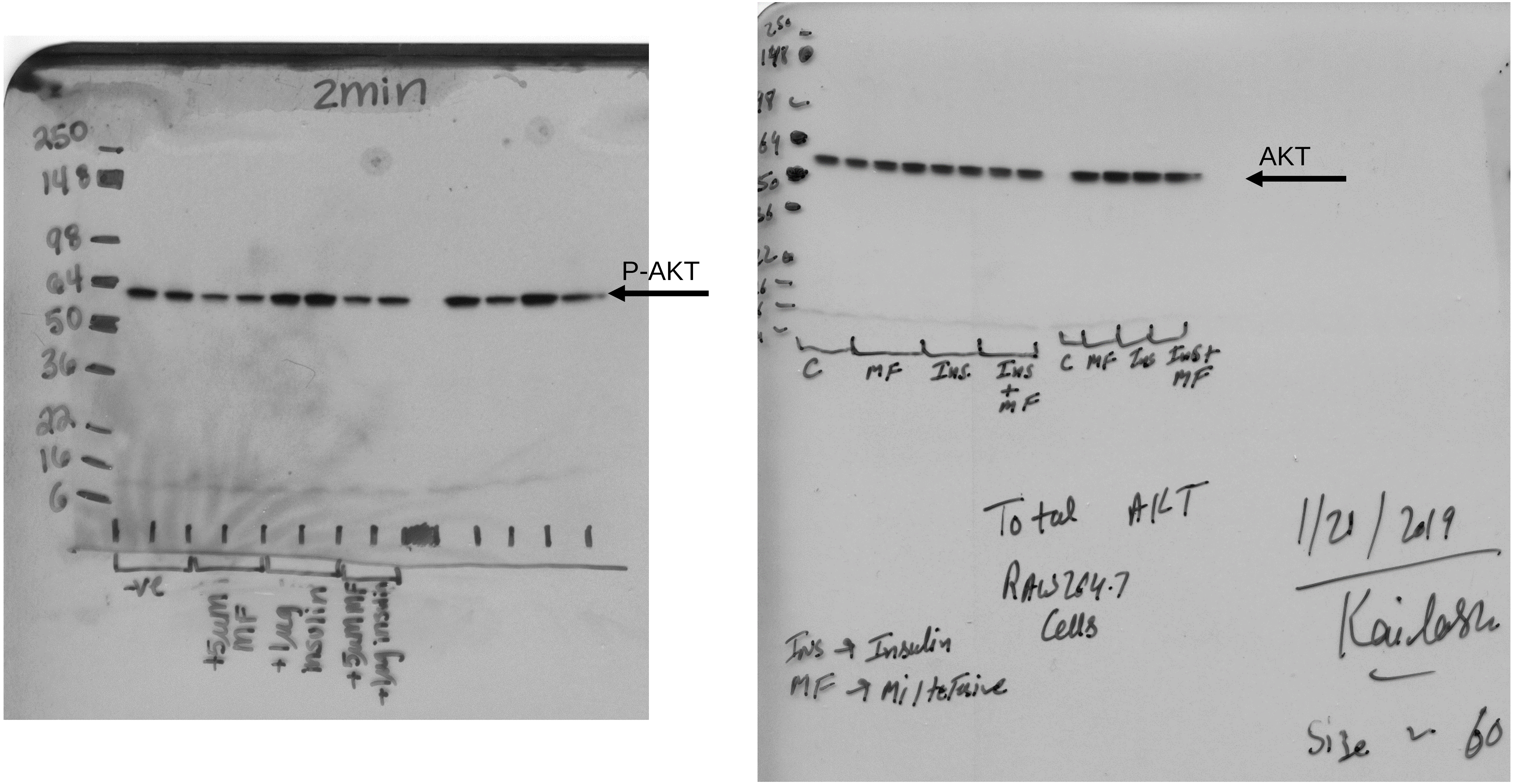

Fig. S16: Full blot of supplementary Figure S8B.

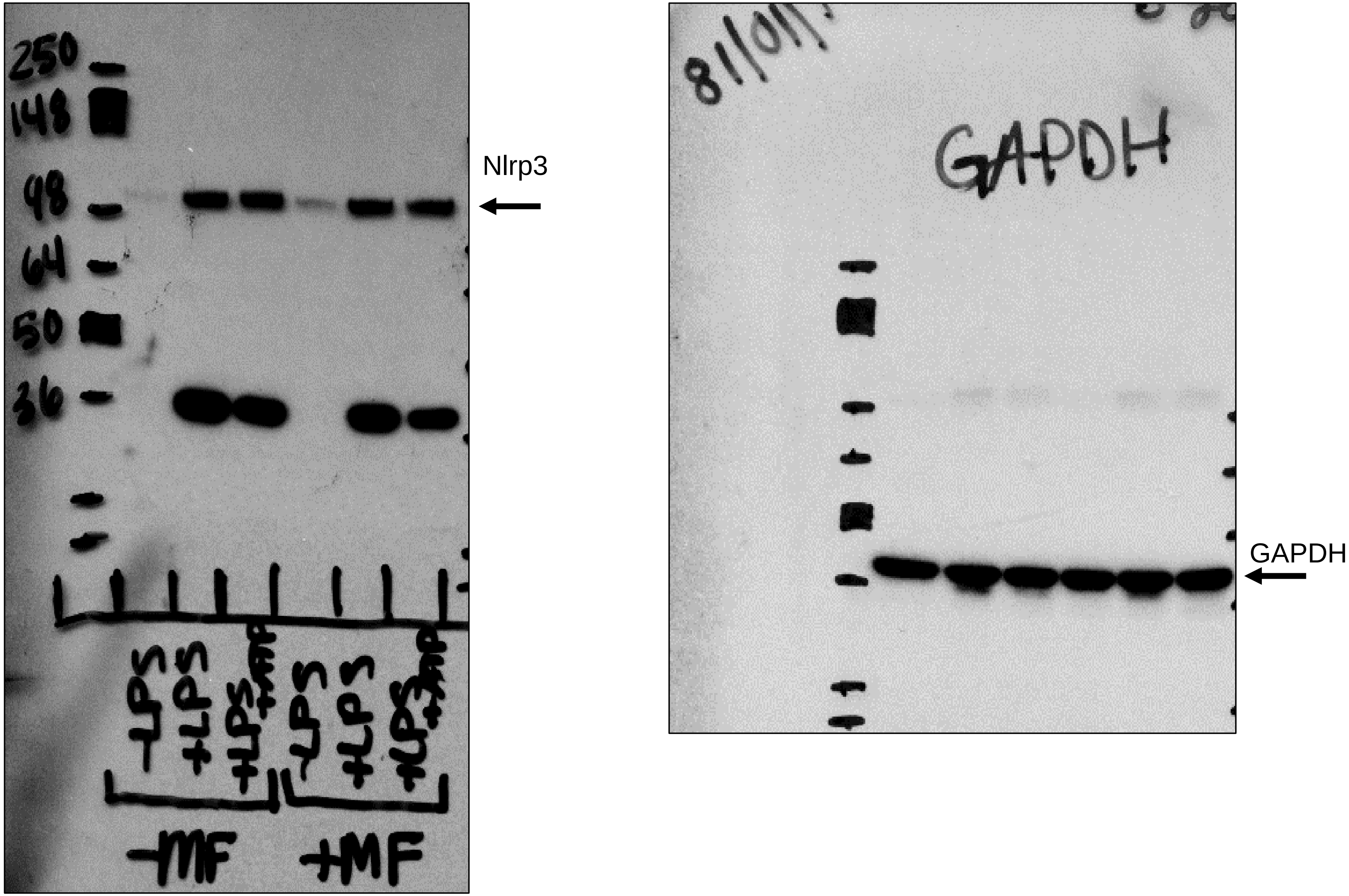

Fig. S17: Full blot of supplementary Figure S8C.

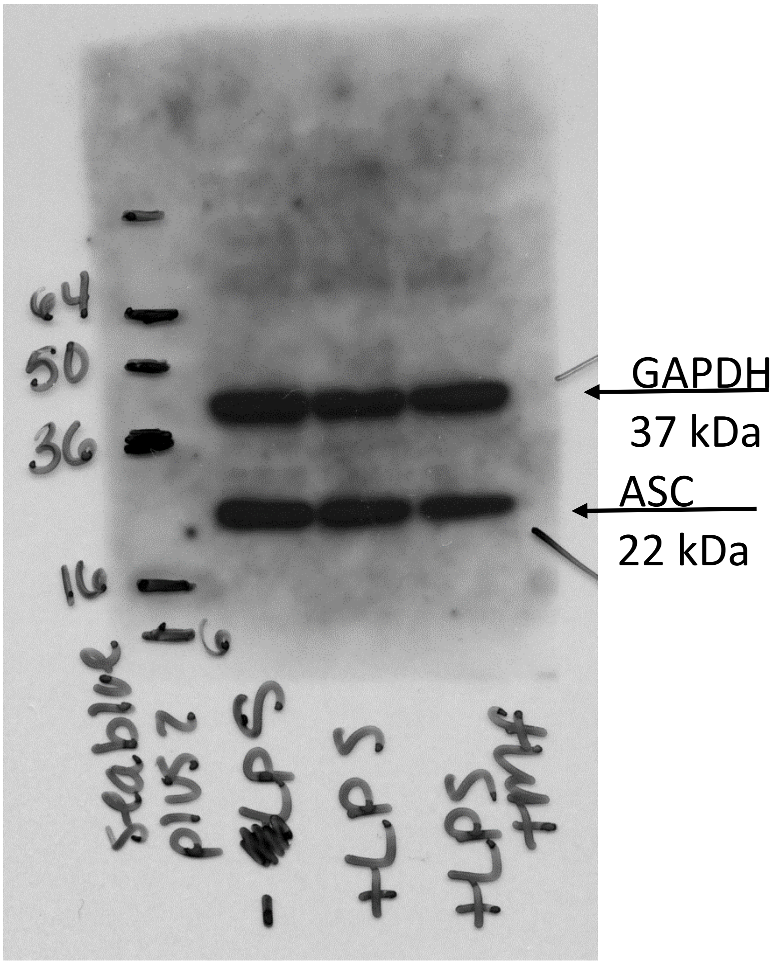

Fig. S18: Full blot of supplementary Figure S8D.

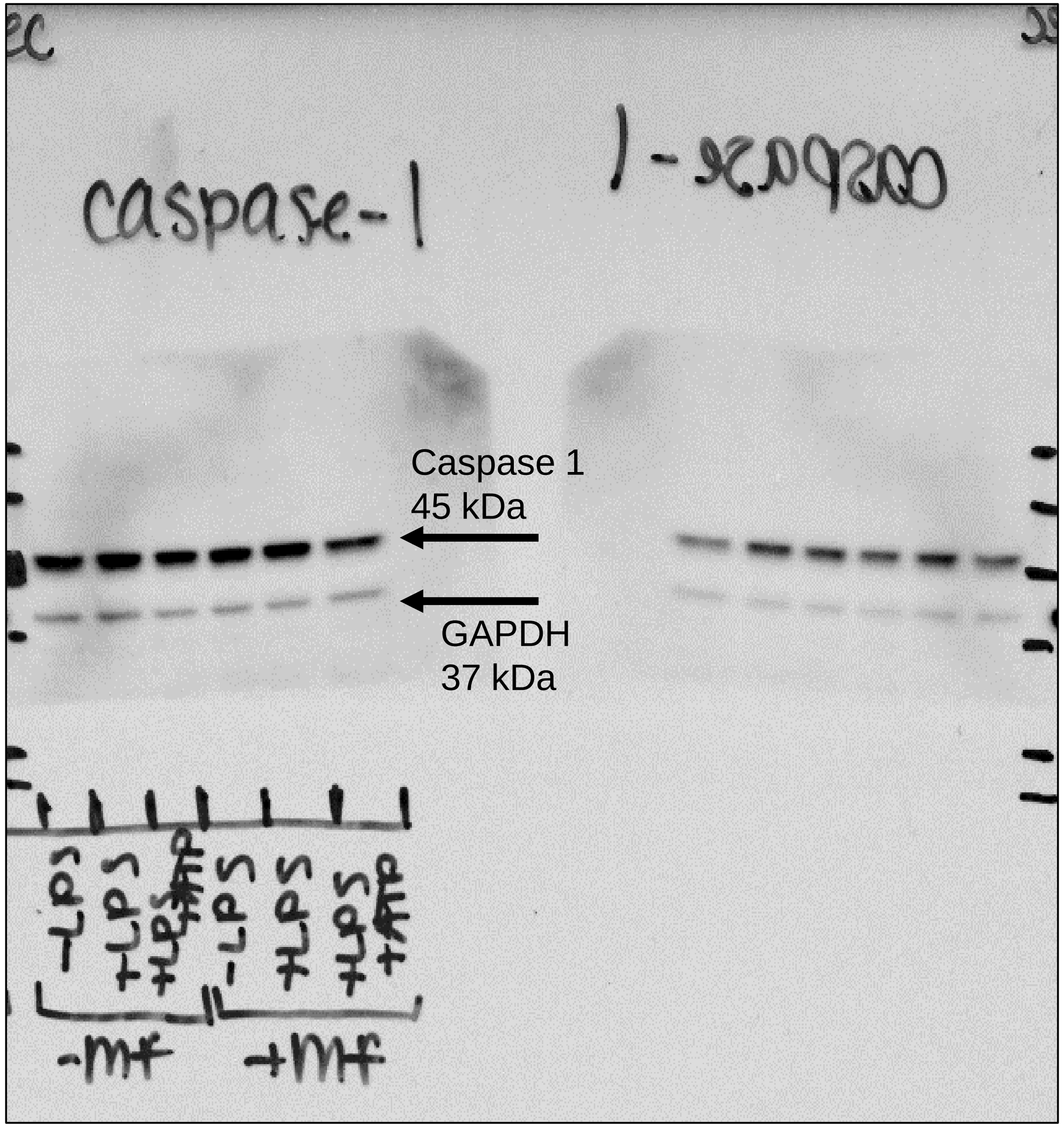

Supplement: Supplementary file 1 — Supplementary Information [file 41598_2019_47610_MOESM1_ESM.pdf]
